# Managements for Obstructive Sleep Apnea in Adults: Review

**DOI:** 10.7759/cureus.9905

**Published:** 2020-08-20

**Authors:** Gantuya Tserenpil, Meklit Gebre, Azka Shahid Zergham, Amanpreet Kaur Sekhon, Bilal Haider Malik

**Affiliations:** 1 Internal Medicine, California Institute of Behavioral Neurosciences & Psychology, Fairfield, USA

**Keywords:** obstructive sleep apnea, sleep disturbance, cpap, oral appliance

## Abstract

Obstructive sleep apnea (OSA) is characterized by recurrent obstruction of the pharyngeal airway during sleep, with resultant hypoxia and sleep fragmentation. It is more common in middle-aged obese men and prevalence is higher in most obese people. However, prevalence is high in African-Americans. OSA is associated with major comorbidities including excessive daytime sleepiness and increased risk of cardiovascular diseases. First and foremost, OSA management starts from educating patients about short-term consequences like motor vehicle accidents, behavioral modifications, long term consequences like cardiopulmonary disease, and resistant high blood pressure. Various types of management options are available for OSA such as weight loss, CPAP, oral appliances, and surgery. The review aims to explain the pathophysiology and cause of the obstruction of the airway in order to choose proper management carefully to decrease the symptoms and cure the disease.

## Introduction and background

Obstructive sleep apnea (OSA) is characterized by episodes of a partial or complete collapse of the upper or lower airway during sleep, resulting in nocturnal hypoxemia [1]. The pathogenesis of OSA is due to the interaction between unfavorable anatomic upper airway (UA) susceptibility and sleep-related changes in UA function [2]. OSA causes excessive daytime sleepiness, negatively affects quality of life, and increases the risk of cardiopulmonary disease, hypertension, and metabolic syndrome [3]. The disease severity is measured using the apnea-hypoxia index (AHI), i.e. the mean number of apneas and hypopneas per hour sleep.

OSA is more common in the middle-aged population with an increased prevalence of obesity [4]. OSA has a significant negative effect on the quality of life and function of the organs and systems, including increased risk of cardiovascular disease, high blood pressure, sexual dysfunction, daytime somnolence, and even sudden death. The other potential consequences of OSA include excessive daytime sleepiness, impaired daytime function, exacerbation of metabolic abnormalities (e.g., impaired glucose tolerance, insulin resistance, type 2 diabetes mellitus, dyslipidemia), and an increased risk of chronic kidney disease and mortality. Several of these conditions appear to have a bidirectional risk association with OSA, including type 2 diabetes mellitus, chronic kidney disease, and left ventricular heart failure [5]. It is also known to be associated with an increased risk of postoperative complications [6].

There are many treatment options to relieve the symptoms and prevent apnea episodes during the night. One of the most important parts of controlling OSA symptoms and maybe a cure is to educate the patient about the risks, consequences, and diet and exercise. Importantly, all patients should be warned about the increased risk of motor vehicle accidents associated with untreated OSA and the potential consequences of driving or operating other dangerous equipment while sleepy. Patients should also be counseled to avoid activities that require vigilance and alertness if sleepy [2]. In addition, continuous positive airway pressure is another effective treatment [7]. There are three major positive airway pressure (PAP) modalities used to treat patients with OSA: continuous positive airway pressure (CPAP), bilevel positive airway pressure (BPAP), and automatic positive airway pressure (APAP). CPAP is generally preferred for most patients because it has been well studied, is simpler to use, and is less costly [5]. Epidemiology, pathophysiology, and treatment options for OSA are reviewed here. The aim of the report is to explore OSA and treatment options that improve the airway and prevent further systemic complications of OSA.

## Review

Prevalence of obstructive sleep apnea (OSA)

Obstructive sleep apnea (OSA) is characterized by episodes of a partial or complete collapse of the upper airway during sleep resulting in nocturnal hypoxemia, a prevalence estimated at 14%-49% in the USA and Europe [8]. Epidemiological studies between 1993 and 2013 show that OSA occurs in 6% of men and in 4% of women in prevalence [1]. However, it is more common in older, obese, males. The prevalence of OSA is common in obese people with Body Mass Index (BMI) >30 and even higher in patients with a BMI >40. The race varies in OSA but there is a slightly increased prevalence in African-Americans [9].

Pathophysiology of OSA

Although the precise pathophysiology of OSA is not well understood, it is believed that the changes in the upper airway mechanics, ventilatory motor output, surrounding tissue structure, and expiratory narrowing triggers the pharyngeal obstruction during sleep in susceptible individuals. Upper airway mechanics - The complete closure of the deformable upper airway due to collapsing transmural pressure during sleep leads to a pathologic increase in the partial pressure of carbon dioxide (PaCO2) in OSA [10]. Ventilatory motor output - changes in the ventilatory drive, including the ventilatory motor output fluctuation during periodic breathing, result in upper airway narrowing and obstruction [3,11,12]. Surrounding tissue - upper airway patency is affected by the passive gravitational forces and high tissue pressure in individuals with mandibular deficiency, large tongue size, fat accumulated in the upper airway, or pharyngeal wall edema secondary to rostral fluid shifts in the recumbent position [13]. Expiratory narrowing - OSA is preceded by expiratory narrowing of the upper airway with increased expiratory resistance or progressive expiratory narrowing [14]. During sleep, there is physiologic hypercapnia (PaCO2 rise by 4-5 mmHg) due to a combination of increased upper airway resistance and decreased ventilatory motor output [15]. The patients with OSA, however, develop considerable hypercapnia and hypoxia due to complete obstruction of the upper airway [1]. OSA is therefore associated with consequences including hypertension, pulmonary hypertension, neurocognitive effects, depressed quality of life, motor vehicle accidents, awakening headache, childhood growth interruption, pregnancy-induced hypertension, fetal growth retardation, and disruption of the patients’ bed-partners’ sleep quality [16].

Managements of OSA

OSA has many different treatment options in order to improve the quality of sleep, eliminate apnea episodes, and increase oxygen saturation during the night [17]. While behavioral modification, CPAP, and oral appliance require long term management, surgery can be done and improve apnea symptoms in days [18]. Effective treatment will reduce many consequences of OSA, such as motor vehicle accidents, cardiovascular mortality, and morbidity. In this section, varies of the managements of OSA will be discussed.

Education and Behavioral Change

Education is one of the utmost important parts of the treatment in patients with OSA. Patients with OSA should be educated about the risks, health consequences, and treatment compliance. Increased risks of motor vehicle accidents are prevalent in patients with OSA. Patients with OSA are 2.5 times more likely to have car accidents due to daytime sleepiness. Patients should be advised to avoid duties such as driving, attentiveness, and surveillance while feeling sleepy. Many behavioral modifications can be combined with treatments such as weight loss, sleeping position, and avoidance of some medications and substances. Obesity is the main risk factor for OSA. In a study of 2148 people with BMI >30, 50% had milder OSA, and 25% showed severe OSA. Prevalence is even higher and reaches 98% in patients with BMI >40 [4,19].

In most cases, weight loss improves the symptoms and cures OSA. Unfortunately, in some cases, weight loss does not bring positive results. However, it is important to modify diet and exercise on a daily basis for patients with OSA [6,20]. Sleeping position is another behavioral change that patients with OSA need to take [21]. Sleeping on the back causes the tongue to relax further to the throat and blocks the airway [22]. Sleeping on the side makes the less obstruction in some patients. Most of the treatments of OSA require long term management that requires daily commitment, so educating patients about the importance of adherence to the treatment is the key to a successful outcome of the treatment and avoids possible negative health consequences [23]. Patients also are encouraged to abstain from alcohol, substance, and some medications such as benzodiazepines. They depress the central nervous system and worsen the drowsiness during the day [2].

Continuous Positive Airway Pressure Therapy

CPAP is one of the most effective therapies used in OSA that opens the airway by delivering the airway through the tube and prevents air collapse during sleep. Positive airway pressure therapy is highly efficacious in reducing car accidents, improving the quality of sleep, reducing blood pressure, and preventing cardiovascular disease development due to OSA [24]. In a meta-analysis of 35 trials, CPAP showed positive results in the apnea-hypopnea index (AHI) mean difference -33.8 events in an hour. However, 10-year randomized trial evidence shows that there is no significant reduction in cardiovascular events [7]. CPAP also decreases the periodic limb movement in mild OSA patients [25]. In another study, CPAP id not directly lower blood pressure and had less effect on it [4,9].

We ran a total of four different trials using the meta-analysis (Table 1) [7-26].

**Table 1:** Meta-Analysis of CPAP therapy outcome CPAP: continuous positive airway pressure

Trials	Purpose	Result
35	reduction in the apnea-hypopnea index	Improved
22	sleepiness, quality of life, cognitive function, and depression	Improved
35	mortality	None
23	motor vehicle crashes	Improved

Choosing the right device is as important as adherence to the daily usage of CPAP. There are three ways of administering the positive airway pressure therapy: continuous positive airway pressure (CPAP), bilevel positive airway pressure (BPAP), and automatic positive airway pressure (APAP) [5,27,28-29].

In Table 2, we discuss different types of positive airway pressure options that can be utilized in OSA management. 

**Table 2:** Continuous airway pressure types CPAP: continuous positive airway pressure, BPAP: bilevel positive airway pressure, APAP: automatic positive airway pressure

Modes	Descriptions	Recommendation
Continuous Positive Airway Pressure (CPAP)	Delivers continuous pressure throughout the respiratory cycle	Mild to moderate sleep apnea, and may benefit cardiogenic and pulmonary edema
Bilevel Positive Airway Pressure (BiPAP)	Presents inspiratory and expiratory airway pressure	Acute hypercapnia, immunosuppressed individuals with infection
Auto-adjusting airway pressure (APAP)	Works via algorithmic control in response to airflow change	Rem-related apnea, and positional apnea

Studies shows that using CPAP for at least four hours a day significantly reduces chronic inflammation and improves daytime sleepiness [27-30].

Oral Appliance

The second most common therapy for OSA is mandibular advancement devices. Two main oral appliances are mandibular advancement splints (MAS) and tongue retaining devices [31]. People may prefer oral appliances over CPAP as they are more comfortable. However, apart from the convenience, oral appliances are proven to be less effective than CPAP, especially in severe OSA. In short-term benefits, MAS reduces the arousal and apnea-hypopnea index [30]. It also improves oxyhemoglobin saturation and snoring. In a study of 10 years follow-up of patients with OSA, MAS presents mean AHI was 31.7±20.6, whereas in the CPAP group 49.2±26.1 in mild to moderate cases [16]. However, MAS shows inconsistent results in terms of complete resolution of OSA and subjective daytime sleepiness improvement [17]. 

Surgery

Surgery for OSA is the last resort treatment [20]. CPAP or oral devices should be tried first. Surgery can be used to eliminate airway obstruction and remove excess tissue from the upper airway without impairing normal functions of the structures to widen the upper airway space [21]. Surgery is recommended in patients with severe OSA (AHI is <20/h), failure of CPAP and other OSA managements, and anatomical location causing obstruction [27]. Contraindications to surgery include patients with dysphagia, morbid obesity, nasopharyngeal reflux, and unstable cardiopulmonary conditions [32]. Many surgical methods can be used to open the airway space for OSA patients, and it is important to evaluate the patients for appropriate surgical procedures to reach the maximum benefit [29-33,34].

In Table 3, we discuss different managements that can be utilized to improve the airway for patients with OSA [5,9,26-29,30,32].

**Table 3:** Different types of surgical options for OSA OSA: obstructive sleep apnea, AHI: apnea-hypoxia index

Methods	Descriptions	Efficacy in OSA
Nasal procedures		
Rhinoplasty	Correct other anatomic nasal airway abnormalities	In case of trauma, narrow bony aperture, or abnormalities that cause blockage of the airway.
Turbinate reduction	Reduce inferior turbinate to reduce airway obstruction	In the turbinate hypertrophy cases, it improves the airway.
Nasal valve surgery	Opens the collapsed valves to increase airway space	
Septoplasty	Strengthens the nasal septum	An only mild case of Obstructive Sleep Apnea (OSA) with curved nasal septum
Upper airway procedures		
Uvulopalatopharyngoplasty	Remove the excess tissue from soft palate, pharynx, and uvula	≥50 percent reduction in the apnea-hypopnea index (AHI) and a postsurgery AHI<20 events per hour
Tonsillectomy	Removes tonsil tissue	216 adult study shows 65% of the AHI mean improvement
Lower airway procedures		
Radiofrequency tongue reduction	Reduces tongue tissue	
Midline glossectomy	Widens the space of hypopharyngeal airspace via carbon dioxide laser	the success rate of 60% reducing AHI
Lingualplasty	Submucosal tongue reduction	In a study, AHI is decreased from 44.0 ± 4.3 to 12.5 ± 2.3.
Global upper airway procedures		
Maxillomandibular advancement	Reposition the upper and lower jaw	21 patients of meta-analysis, it shows an improvement of 86% in AHI

## Conclusions

In this review article, possible management of OSA and outcomes are discussed in detail. Many articles associated with OSA management, pathophysiology, prevalence, outcomes of the treatments are summarized and reviewed here. One of the most important parts of controlling OSA symptoms and maybe a cure is to educate the patient about the risks, consequences, and diet and exercise. CPAP is one of the promising treatments if the patient is compliant with the daily regime. The second efficient therapy is oral devices because of their convenience to use over CPAP. Finally, we discussed various surgical procedures that can be done in OSA if other managements are not efficient. Understanding the pathogenesis of the OSA and choosing the right treatment depends on the cause of obstruction, and it is the key to a successful outcome of the management. 
